# A global priority: addressing violence against children

**DOI:** 10.2471/BLT.19.247874

**Published:** 2021-03-21

**Authors:** Yusra Ribhi Shawar, Jeremy Shiffman

**Affiliations:** a Johns Hopkins University, Bloomberg School of Public Health, Paul H. Nitze School of Advanced International Studies, 615 N Wolfe Street, Baltimore, MD 21025, United States of America.

## Abstract

**Objective:**

To determine the reasons for the lack of priority given to addressing violence against children, and to identify the challenges that proponents must address to improve prioritization of this issue.

**Methods:**

We reviewed relevant literature to identify experts to interview. We carried out a thematic analysis of the literature and interview transcripts. We iteratively developed data coding on the many characteristics of violence against children, on the framing of the issue by proponents, and on the problem of governance – that is, how proponents organize themselves for collective action.

**Findings:**

The analysis of our data sources reveals many obstacles for global prioritization of addressing violence against children, including the forms of violence considered, inadequate data to describe prevalence and a lack of evidence of the effectiveness of proposed solutions. There exists fundamental disagreement among proponents on the recently introduced frame of violence against children, including differences in the types of violence that should be prioritized and in the proposed solutions (e.g. prevention or remediation). On governance, competition between networks focused on specific forms of violence is hampering efforts to create strong governing institutions.

**Conclusion:**

Despite the complex challenges identified, proponents have made some progress in global prioritization of addressing violence against children. To improve this prioritization further, proponents must resolve framing tensions and strengthen governance mechanisms to promote shared goals, while ensuring that networks focused on particular forms of violence are able to maintain their distinct identities.

## Résumé

**Objectif:**

Définir les raisons expliquant pourquoi la violence envers les enfants n'est pas une priorité, et identifier les défis que les acteurs de cette lutte doivent relever pour améliorer sa visibilité.

**Méthodes:**

Nous avons effectué une revue documentaire afin de trouver des experts à interroger. Nous avons également réalisé une analyse thématique de la littérature et des transcriptions d'entretiens. Nous avons développé sous forme itérative un codage des données reprenant les nombreuses caractéristiques de la violence envers les enfants, la formulation des enjeux par les acteurs de la lutte, et le problème de la gouvernance. En d'autres termes, comment ces acteurs s'organisent pour mener des actions collectives.

**Résultats:**

L'analyse de nos sources de données révèle de multiples obstacles dans la gestion mondiale des priorités inhérentes à la lutte contre la violence envers les enfants, parmi lesquels les types de violences pris en compte, l'inadéquation des données décrivant leur prévalence, et le manque de preuves d'efficacité des solutions proposées. Il existe des désaccords profonds parmi les acteurs de la lutte sur le cadre récemment instauré pour définir la violence envers les enfants, notamment des divergences au niveau des types de violences considérées comme prioritaires et des solutions proposées (par exemple la prévention ou la réparation). Quant à la gouvernance, la concurrence que se livrent les réseaux dédiés à des violences spécifiques nuit aux efforts de création d'institutions gouvernementales solides.

**Conclusion:**

Malgré la complexité des défis identifiés, les acteurs de la lutte contre la violence envers les enfants ont accompli quelques progrès dans l’établissement des priorités en la matière. Pour poursuivre sur cette lancée, ils doivent résoudre les tensions que suscite le cadre de référence et renforcer les mécanismes de gouvernance afin de promouvoir des objectifs communs, tout en veillant à ce que les réseaux dédiés à des violences spécifiques puissent conserver leur identité propre.

## Resumen

**Objetivo:**

Determinar las razones por las que no se da prioridad al tratamiento de la violencia contra los niños, e identificar los retos que los defensores deben abordar para mejorar la priorización de esta cuestión.

**Métodos:**

Revisamos la bibliografía pertinente para identificar a los expertos a los que entrevistar. Realizamos un análisis temático de la bibliografía y de las transcripciones de las entrevistas. Desarrollamos de forma iterativa la codificación de los datos sobre las múltiples características de la violencia contra los niños, sobre la estructuración de la cuestión por parte de los defensores y sobre el problema de la gobernanza, es decir, cómo se organizan los defensores para la acción colectiva.

**Resultados:**

El análisis de nuestras fuentes de datos revela muchos obstáculos para la priorización global del tratamiento de la violencia contra los niños, incluyendo las formas de violencia consideradas, datos inadecuados para describir la prevalencia y la falta de pruebas de la eficacia de las soluciones propuestas. Existe un desacuerdo fundamental entre los defensores sobre el marco recientemente introducido de la violencia contra los niños, incluidas las diferencias en los tipos de violencia que deben priorizarse y las soluciones propuestas (por ejemplo, prevención o remedio). En cuanto a la gobernanza, la competencia entre redes centradas en formas específicas de violencia está obstaculizando los esfuerzos por crear instituciones de gobierno fuertes.

**Conclusión:**

A pesar de los complejos desafíos identificados, los defensores han realizado algunos progresos en la priorización global del tratamiento de la violencia contra los niños. Para mejorar aún más esta priorización, los defensores deben resolver las tensiones de encuadramiento y fortalecer los mecanismos de gobernanza para promover los objetivos compartidos, asegurando al mismo tiempo que las redes centradas en formas particulares de violencia puedan mantener sus identidades distintivas.

## ملخص

الغرض تحديد أسباب عدم منح الأولوية لمواجهة العنف ضد الأطفال، وتقرير التحديات التي يجب على المؤيدين مواجهتها لتحسين أولوية هذه القضية.

الطريقة قمنا بمراجعة المنشورات ذات الصلة لتحديد خبراء لإجراء مقابلات معهم. قمنا بتحليل موضوعي للمنشورات ونصوص المقابلات. قمنا بوضع ترميز للبيانات بشكل متكرر حول العديد من خصائص العنف ضد الأطفال، وحول وضع إطار للقضية بواسطة المؤيدين، وحول مشكلة الحوكمة، أي كيفية تنظيم المؤيدين لأنفسهم في العمل الجماعي.

النتائج يكشف تحليل مصادر البيانات لدينا عن العديد من العوائق التي تواجه وضع أولوية عالمية لمواجهة العنف ضد الأطفال، بما في ذلك أشكال العنف المنظورة، والبيانات غير الكافية لوصف مدى الانتشار، وعدم وجود أدلة على فعالية الحلول المقترحة. هناك خلاف أساسي بين المؤيدين حول الإطار المطروح مؤخرًا للعنف ضد الأطفال، بما في ذلك الاختلافات في أنواع العنف التي يجب أن تُمنح الأولوية، وفي الحلول المقترحة (مثل الوقاية أو العلاج). وبخصوص الحوكمة، فإن المنافسة بين الشبكات والتي تركز على أشكال محددة من العنف، تعوق جهود إنشاء مؤسسات حوكمة قوية.

الاستنتاج على الرغم من التحديات المعقدة التي تم تحديدها، إلا أن المؤيدين قد حققوا بعض التقدم في تحديد أولوية عالمية لمواجهة العنف ضد الأطفال. لتحسين هذه الأولوية بشكل أكبر، يجب أن يقوم المؤيدون بحل توترات التأطير، وتقوية آليات الحوكمة لتعزيز الأهداف المشتركة، مع ضمان أن الشبكات التي تركز على أشكال معينة من العنف قادرة على الحفاظ على هوياتها المحددة. ****

## 摘要

**目的:**

确定对解决暴力侵害儿童问题缺乏优先考虑的原因，并确定支持者为改善这一问题的优先次序必须解决的挑战。

**方法:**

我们查阅了相关文献评论以确定访谈需要邀请的专家，然后对文献和访谈记录开展了主题分析。经过反复测试，，我们对儿童遭受暴力侵害的特征、支持者对该问题的界定，以及治理问题进行了数据编码，研究支持者应如何组织开展集体行动。

**结果:**

我们对我们的数据来源进行了分析，揭示了在全球优先处理暴力侵害儿童问题上的许多障碍，包括所考虑的暴力形式、描述普遍程度的数据不足，以及缺乏证据证明所提议解决方案的有效性。支持者对最近提出的如何界定对儿童的暴力存在根本分歧，包括在应列为优先事项的暴力类型和拟议的解决办法（例如预防或补救）方面存在差异。在治理方面，专注于不同暴力形式的不同组织网络之间所存在的竞争关系，阻碍着建立强大的治理机构这一目标的实现。

**结论:**

尽管存在一些严峻的挑战，但支持者在全球优先处理暴力侵害儿童问题方面已经取得了一些进展。为了进一步改善该问题的优先级别，支持者必须解决在该问题界定方面的紧张关系，加强治理机制，以促进实现共同目标，同时确保专注于特定暴力形式的不同组织网络能够坚守他们各自的准则。

## Резюме

**Цель:**

Определить причины недостаточного внимания, уделяемого решению проблем насилия в отношении детей, и задачи, которые необходимо решать инициаторам мер для повышения приоритетности этого вопроса.

**Методы:**

Авторы изучили соответствующую литературу с целью отбора экспертов для проведения интервью. Был проведен тематический анализ литературы и стенограмм интервью. Авторы итеративно разрабатывали алгоритмы кодирования данных по многим характеристикам видов насилия в отношении детей, по формулировке проблемы инициаторами мер, а также по проблеме организации управления, то есть по тому, как инициаторы мер организуют себя для коллективных действий.

**Результаты:**

Анализ источников данных показывает множество препятствий на пути к установлению глобальных приоритетов в борьбе с насилием в отношении детей, включая рассматриваемые формы насилия, недостаточность данных для описания их распространенности и отсутствие доказательств эффективности предлагаемых решений. Среди инициаторов мер существуют серьезные разногласия по поводу недавно введенной классификации видов насилия в отношении детей, включая различия в видах насилия, которым следует уделять первоочередное внимание, и предлагаемых решениях (например, предотвращение или устранение последствий). Что касается организации управления, конкуренция между сетями, ориентированными на борьбу с конкретными формами насилия, препятствует усилиям по созданию сильных институтов управления.

**Вывод:**

Несмотря на выявленные сложные проблемы, инициаторам мер удалось добиться определенного прогресса в определении глобальных приоритетов в борьбе с насилием в отношении детей. Для дальнейшего совершенствования приоритизации инициаторы мер должны устранить структурные разногласия и укрепить механизмы управления для содействия достижению общих целей, при этом гарантируя, что сети, ориентированные на борьбу с конкретными формами насилия, останутся неизменными.

## Introduction

Every year, approximately three quarters of the world’s children (those aged 0–19 years) experience some form of violence;^1^ this proportion may have grown even larger as a result of the ongoing coronavirus disease 2019 (COVID-19) pandemic.^2^^–^^6^ An early experience of violence increases the risk of a child perpetuating violence later in life, and is a contributing factor in a wide range of high-risk behaviours, mental health problems and physical morbidity across the life course.^7^ The global economic costs of violence against children are also high, recently estimated to be as much as 7 trillion United States dollars.^8^

Despite the enormity of this problem, addressing violence against children is a low priority for global organizations and governments and receives minimal investment.^9^^,^^10^ Social science research indicates that two factors exist which influence the priority received by an issue: (i) the issue characteristics;^11^^–^^14^ and (ii) proponent capacities, especially as they concern framing and governance.^12^^,^^15^^–^^19^ First, an issue is more likely to receive priority if: the affected population is perceived sympathetically and is politically powerful;^11^ it involves bodily harm; specific actors can be assigned blame; the severity of the issue is backed by reliable data;^12^ and interventions are perceived as cost-effective, simple to implement and backed by scientific evidence.^13^^,^^14^ Second, an issue is more likely to receive priority if proponents frame the issue effectively (i.e. agree on the nature of the problem and its solutions, and present the issue in ways that resonate with policy-makers) and can build successful coalitions to facilitate collective action.^18^^,^^19^

By conducting a review of relevant organizational reports and peer-reviewed publications, and by interviewing experts in the field of violence against children, we amassed a large collection of both published data and interview transcripts. We triangulated among data sources to extract and verify information on major developments, aiming to determine the reasons for the ongoing lack of priority given to addressing violence against children.

## Methods

### Literature review

We searched for relevant publications on Google Scholar, ProQuest and JSTOR databases, and on the websites of organizations concerned with violence against children. We restricted our search to literature published in English between the years 1989 and 2020, and included organizational reports and peer-reviewed research articles on addressing or preventing violence against children (a list of search terms is available in the data repository).^20^

### Interviews

Employing a purposive sampling strategy, we identified potential interviewees through our literature review and by asking informants whom they considered to be most centrally involved in the field of violence against children. We contacted 39 experts, and obtained the agreement of 28 (response rate: 71.8%). The informants’ affiliations are listed in Box 1. In Box 2, in which we assign our informants an identification number of 1–28, we also highlight the wide range of primary affiliation types in both high-income and low- to middle-income countries.

Box 1 **Organizational affiliations of 28 key informants^a^ interviewed in study of global priority given to addressing violence against children, 2018–2020**

1000 Days; Alliance for Early Success; Amnesty International USA; Bernard van Leer Foundation; Buffett Early Childhood Fund; Center on Child Protection and Wellbeing Universitas Indonesia (PUSKAPA); Center on International Cooperation, New York University; Centers for Disease Control and Prevention; *Child Abuse and Neglect*;^b^ *Child Maltreatment*;^b^ Child Protection in Crisis Learning Network; Child Protection Unit Network; Children and Violence Evaluation Challenge Fund; Children’s Rights and Violence Prevention Fund; Columbia University; Early Opportunities Limited Liability Co.; Elevate Children Funders Group; Every Child Protected Against Trafficking (ECPAT) International; Global Alliance on Reporting Progress on Promoting Peaceful, Just and Inclusive Societies; Global Leaders for Young Children; Global Partnership to End Violence against Children; Harvard University; Ignite Philanthropy; International Committee of the Red Cross; International Society for the Prevention of Child Abuse and Neglect; Know Violence in Childhood Global Initiative; McGill University; Oak Foundation; Open University of Sri Lanka; Promundo; Raising Voices; Save the Children; *Sexual Abuse: A Journal of Research and Treatment*; Together for Girls; Union Bank of Switzerland Optimus Foundation; United Nations Foundation; United Nations International Children’s Emergency Fund; United States Department of Health and Human Services; University of Cape Town; University of New Hampshire; University of the Philippines Manila; University of the West of England; Wellspring Advisors; World Bank; World Health Organization; World Health Organization’s Violence Prevention Alliance

^a^ Some of the 28 key informants have multiple affiliations.^b^ Affiliations formatted in italics are journals.

Box 2 **Types of primary organizational affiliation of 28 key informants interviewed in study of global priority given to addressing violence against children, 2018–2020**

**High-income country**

Interviewee 1: academic; 2: local; 3: global alliance/initiative; 4: foundation; 6: academic; 7: global alliance/initiative; 9: academic; 10: international; 11: national; 12: global alliance/initiative; 14: international; 16: foundation; 21: foundation; 22: global alliance/initiative; 24: foundation; 25: international; 26: international; 27: foundation; 28: global alliance/initiative.

**Low- to middle-income country**

Interviewee 5: national; 8: academic; 13: academic; 15: local; 17: local; 18: academic; 19: academic; 20: national alliance/initiative; 23: global alliance/initiative.

We conducted our interviews, each of which lasted an average of 1 hour, by Skype (Microsoft Corporation, Redmond, United States of America, USA) between June 2018 and November 2020. With the permission of key informants, we recorded and manually transcribed all interviews. While we asked most informants the same initial questions (such as the extent to which they perceived addressing violence against children to be a global priority), we personalized many interview questions according to the background of the particular participant. An outline of the interview structure is available in the data repository.^20^

### Analysis

We redacted all interview transcripts and notes to ensure the anonymity of informants. We sought to minimize bias by triangulating among data sources, and compared interview transcripts both between informants and with the collected literature. 

We used an iterative process to develop the data coding. We began by coding data by (i) issue characteristics and (ii) proponent capacity, and this evolved as we collected additional data. Within issue characteristics, we added subcodes for: forms of violence, children’s powerlessness, inadequate data, interventions and the availability of solutions. The complex characteristics of the issue generate disagreement between proponents about how it should be understood and addressed (a framing challenge) and how to generate collective action (a governance challenge). We therefore added subcodes for: the acceptability of the framing of violence against children, disagreements concerning solutions and positioning difficulties within framing; and fragmentation, leadership uncertainties and coalition-building difficulties within governance. 

We used the Consolidated Criteria for Reporting Qualitative Research guidelines to ensure comprehensive reporting of our data collection and analysis processes.^21^

### Ethics

The study obtained ethical approval from the Institutional Review Board of the American University (Washington, DC, USA).

## Results

### Issue characteristics

We observed several features of the issue of violence against children that should attract global political support, including: the high global prevalence of violence against children;^22^ the fact that it is a risk factor for a wide range of problems; and its unacceptability in most societies (key informant 2).^23^ However, our analysis also revealed many difficulties posed by the nature of the issue.

#### Forms of violence

Violence against children has many forms and is inflicted by a wide array of perpetrators, making the issue difficult for policy-makers to understand and address.^24^ The violence can involve neglect or abuse, which can be physical, emotional or sexual. The issue also encompasses a broad range of behaviours, including exposing a child to prostitution, physical beating or child labour.

#### Children’s powerlessness

Several respondents highlighted how children lack power to sway decision-makers and gain access to protective structures (informants 19, 20 and 23). The most disempowered youth are disproportionately targeted, and the perpetrators of violence against children are often caregivers.^25^ Reflecting on these difficulties, informant number 23 commented: “We do cherish children…but there is also an element of: Do their rights come second to mine as a parent or as an adult? Something there has to shift.”

Other informants (27 and 28) reported that measures necessary to contain the COVID-19 pandemic have further isolated children from protective structures, increasing their risk of exposure to violence.^26^ Informant number 27 noted: “The protective ecosystem for children – the teachers, police officers, social workers – are no longer there to track and have contact with kids… Schools in many ways were safe havens.”

#### Inadequate data

Many informants (1, 2, 4, 9, 11, 20 and 27) reported how insufficient and unreliable data are another problem. On the impact on advocacy, informant number 20 noted: “At the Ministry of Education they can say: ‘This is what the problem is. There are X number of children who are not in school.’ You can’t say that in violence prevention work.”

International analyses of the prevalence of violence against children have grown significantly in the last decade,^27^^,^^28^ bolstered by the global school-based student health survey,^29^ a World Health Organization (WHO) and United States Centers for Disease Control and Prevention collaboration, and the Violence Against Children Surveys that were first conducted in 2007.^30^ Historically, however, many epidemiological studies on violence against children have lacked methodological rigour, focused on high-income countries and produced imprecise data.^31^

#### Intervention complexity 

The complexities of preventing and addressing the problem of violence against children complicate efforts to convince policy-makers that it is surmountable. Many of our informants (1, 2, 4, 12, 18 and 22) described how addressing violence against children requires the involvement of many fields, including health, child protection, education, social welfare and justice.^23^ Our analysis revealed that violence against children is considered intergenerational, fuelling the perceptions of policy-makers of its inevitability (informants 2, 3, 11 and 22).^32^

#### Unproven solutions

Our informants (1, 9, 15 and 22) also commented on the additional problem of the lack of evidence of the effectiveness of proposed interventions. The *Ending violence in childhood* report^1^ presents the latest evidence of the causes and consequences of violence in childhood, and offers ideas on how such violence can be prevented.^33^^,^^34^ Another promising development discussed (informants 8, 11, 14, 23 and 27) is the launch of INSPIRE, a package of seven evidence-based strategies to prevent violence against children (implementation and enforcement of laws; norms and values; safe environments; parent and caregiver support; income and economic strengthening; response and support services; and education and life skills).^24^ Key informant number 14 reflected on its impact on advocacy: “INSPIRE is probably the best example of things which have an evidence base about what you can do.” However, even some of the promising interventions, such as home visitation^35^ and microfinance initiatives,^36^ do not definitively prevent the reoccurrence of violence against children. Informant number 10 described the situation as one in which “interventions [and] policies have run way, way ahead of the evidence.”

### Framing

#### Framing acceptability 

Our analysis indicated that a central point of contention is the acceptability of the recently emerged violence against children frame. This frame sits uneasily with the longer-standing child protection frame, historically associated with the United Nations International Children’s Emergency Fund (UNICEF) and other organizations, which identifies social work as the lead profession (informants 3, 9, 10, 11, 13 and 24).^37^ The violence against children frame calls for the application of multidisciplinary approaches and evidence-based practices to prevent multiple forms of violence affecting children (informants 3 and 10). Acceptance and use has grown over the last decade, and recent initiatives and policy developments using the frame include: the sustainable development goal (SDG) target of ending violence against and torture of children; the INSPIRE strategies;^24^ the Violence Against Children Surveys;^30^ the establishment of the Global Partnership to End Violence Against Children; and the appointment of a United Nations (UN) Special Representative of the Secretary-General on Violence Against Children.

The new violence against children frame elicits divergent reactions. Some see the shift in frame as enabling space for greater multidisciplinary engagement, attracting previously uninvolved actors such as WHO and the United States Centers for Disease Control and Prevention, and bringing greater focus to advocacy efforts than the broader child protection frame (informants 4, 9, 12–14, 22 and 23).^38^ Others (informants 1, 17, 19, 20 and 24), particularly social workers, prefer to describe their work as falling within the realm of a particular sector (e.g. child protection), discipline (e.g. social work; informant 20) or type of violence (e.g. sexual; informant 9). Many working at national and subnational levels also find that the violence against children frame does not resonate (informants 1, 9, 18, 21, 22).

Even among those embracing the violence against children frame, there exist disagreements about which types of violence should be prioritized (informants 3 and 9–12). For example, one disagreement is around the extent to which corporal punishment and sex trafficking should be included (informants 2, 10 and 11); some view the cultural and contextual differences in the case of corporal punishment, and sensationalization with respect to sex trafficking, as potentially reducing priority to address other forms of violence against children. Informant 10 described how “different constituencies push their particular type of violence…distorting the evidence in any way they can to get more attention and more money.”

#### Disagreements concerning solutions

Our analysis indicated that proponents also diverge on whether the focus should be on violence against children prevention or remediation (informants 2, 4, 5, 9–11, 14, 17 and 20). Many working in law enforcement emphasize the need for stronger legislation and prosecution, while those working in public health instead argue for education and health programmes to effect social change (informant 1). Further tension among proponents results from disagreements in the perception of what is evidence-based, and whether interventions should be integrated into existing programmes (e.g. paediatrics) or form stand-alone programmes.^23^ Informants also differed in their opinion about the extent to which interventions should be targeted towards boys in addition to girls (informants 5, 9, 10 and 17), as some were concerned about the gross neglect of sexual violence against boys.^39^

Several informants noted how the INSPIRE strategies and an End Violence Solutions Summit in 2018^40^ were critical for building consensus among proponents (informants 3, 5 and 12). Nevertheless, INSPIRE has also garnered criticism: some claim it overstates the evidence and relies excessively on data from the gender-based violence field (informant 26). Critics also report that the experiences and voices of local actors are not sufficiently integrated (informants 9 and 26). Recognizing these criticisms, WHO, the Global Partnership to End Violence Against Children and others have begun supporting five low- and middle-income countries to implement INSPIRE strategies at scale (informant 27).

#### Positioning difficulties

Our analysis indicated that differences surrounding problem definition and proposed solutions have made it difficult for proponents to advance a case that may motivate political leaders to act. One difficulty is the tendency of proponents to emphasize problems rather than solutions (informants 2, 3, 5, 14 and 22).^10^^,^^41^ Our respondents noted the perpetuation of a child-misery narrative that dismisses the dignity, resilience and agency of a child (informants 3, 5 and 18). Informant 5 remarked: “I think the default for many years has been the…selling of children’s suffering…This leads to re-victimization and portraying girls and children as hopeless and without agency.”

### Governance

#### Fragmentation

Our analysis revealed how violence against children encompasses distinct networks that may have no strong motivation to cooperate. Some networks address specific forms of violence, such as the Global Initiative to End All Corporal Punishment of Children. Others focus on particular target populations, such as the Alliance for Child Protection in Humanitarian Action. Proponents are also divided by sector, including education, social work, public health and law enforcement (informants 1, 6, 7 and 18). Compounding this fragmentation is the multitude of actors working on the issue at a global level (informants 5, 7 and 10) and the multiple campaigns addressing violence. Some of our informants (3, 12 and 21–24) perceived turf battles to be rampant, with contention over control of the agenda and a desire to gain credit for their contributions. While the growing number of actors concerned with violence against children is advantageous, it creates difficulties in advancing a common strategy.

This fragmentation prompted our informants to question whether violence against children even constitutes a cohesive field. While some affirm the existence of a violence against children field (informant 11), others view it as a loose set of networks (informants 8–10 and 19). Respondents also reported a disjointedness between those working on violence against children at the global and the local level, perceiving the issue to be a donor-driven agenda (informants 9, 17, 21 and 22).

#### Leadership uncertainties

Individual and organizational leadership has also been a challenge (informants 3, 10, 14 and 21). Several respondents reported that WHO, UNICEF and the United States Centers for Disease Control and Prevention are central players in elevating the agenda for addressing violence against children; these organizations have coordinated several forums aimed at bringing proponents together and disseminating knowledge and experience of the issue. However, respondents reported that these and other forums attract only a subset of proponents (informants 1, 9, 10 and 13).

Many informants are hopeful about the Global Partnership to End Violence Against Children, which aims to scale up action to protect children through implementation of the INSPIRE strategies. The idea for the partnership emerged in 2014 as proponents came together to lobby for the inclusion of violence against children in the SDGs (informant 2). Since its launch, the partnership has attracted 26 path-finding countries (informant 12). An associated, multi-donor Fund to End Violence Against Children was also created. In 2020, the partnership organized a leaders’ statement,^42^ which brought together the heads of major UN, international and civil society organizations in a joint statement on protecting children from violence and reducing the impact of COVID-19 on children (informants 27 and 28). Respondent 12 commented: “The way [the partnership] is positioned, with one foot in the UN and one foot outside, it’s really powerful…it plays the UN card, which gets it the highest-level political access it needs, but it doesn’t have to play by all UN rules.” Nonetheless, there remain concerns regarding its ability to balance the agendas of involved constituencies (informants 2, 3 and 12) and its perceived threat to the power of long-standing actors in this area (informant 12).

#### Coalition-building complexities

Several proponents recognize the need to build alliances with organizations working in more powerful sectors outside the violence against children field (informant 3).^10^ However, several factors have made coalition-building difficult, including the fragmentation of proponents and their historical lack of interest in engaging with other communities. The lack of linkages with violence against women and early childhood development – the most obvious allies for addressing violence against children – are particularly notable (informants 1, 8, 10, 13, 15 and 27).^43^ Some proponents are concerned about their issue becoming diluted if integrated with issues of greater prominence. This ambivalence also flows in the other direction (informants 2, 3, 5, 8, 9, 15, 20 and 21); proponents concerned with violence against women fear that linking the two issues would negatively affect available resources (informants 2, 9, 18 and 26). Despite the limited success of those concerned with violence against children in recruiting allies, there are several encouraging developments and opportunities for strengthening collaboration efforts, especially with early childhood development, violence against women and, more recently, positive parenting. These include the Global Plan of Action to strengthen the role of the health system to address interpersonal violence against women and girls, and against children.^44^^,^^45^

## Discussion

Despite the challenges of the unfavourable characteristics of the issue of addressing violence against children, and the difficulties in achieving agreement among individuals and organizations on framing and governance, several recent positive developments may support improved global prioritization for the issue. These developments include the establishment of important partnerships, funds and surveys; recognition of the issue within the SDGs; rising national interest in addressing the problem; opportunities for collaboration with communities working on violence against women and early childhood development; the 2020 publication of the *Global status report on preventing violence against children*; and the highlighting of the issue by the COVID-19 pandemic.^42^^,^^44^^,^^46^

However, proponents face at least three strategic challenges as they seek to augment global prioritization of the issue. The first is to find means of strengthening global governance of the issue, in ways that will facilitate a growing acceptance of the violence against children frame, while ensuring that actors that focus on particular forms of violence do not perceive the undermining of their missions. The Partnership to End Violence Against Children already embraces such an umbrella strategy. 

The second challenge is to find opportunities to graft the issue onto other agendas already prioritized in the SDGs, and to take advantage of the attention brought to the issue by the COVID-19 pandemic. 

Finally, the third challenge is to work towards reversing the perceptions held by many policy-makers concerning the intractability of the problem, by moving from a focus on the gravity of the situation to an emphasis on its solubility. Emergent consensus among proponents on strategies, as evidenced by the INSPIRE package, the End Violence Solutions Summit and growing research on the cost–effectiveness of interventions to prevent violence, highlights the opportunities that exist to re-frame the issue.

A limitation of our study is that few of our respondents represented the education and law and justice sectors, and none were affiliated to an organization representative of those fields at the time of interview (e.g. the UN Educational, Scientific and Cultural Organization and the UN Office on Drugs and Crime). We made efforts to account for these perspectives in the literature review by searching for relevant documents published by organizations dedicated to these sectors.

Our study has revealed that there exists a complex web of reasons to explain the current lack of global priority given to addressing violence against children. To improve this prioritization, proponents must resolve framing tensions and strengthen governance mechanisms to promote shared goals, while ensuring that networks focused on particular forms of violence have the opportunity to maintain their distinct identities. 
